# Exploring access to desired and appropriate contraception during incarceration: A qualitative description of the experiences of women in custody in Ontario, Canada

**DOI:** 10.1177/17455057261432612

**Published:** 2026-03-13

**Authors:** Emily Norris, Jessica Gaber, Julia Zhu, Lindsay Jennings, Meredith Vanstone, Jessica Liauw, Jessica Jurgutis, Katherine E. McLeod, Fiona G. Kouyoumdjian

**Affiliations:** 1 Department of Obstetrics and Gynecology, McMaster University, Hamilton, ON, Canada; 2 Department of Family Medicine, McMaster University, Hamilton, ON, Canada; 3 Faculty of Health Sciences, McMaster University, Hamilton, ON, Canada; 4 Department of Health Research Methods, Evidence, and Impact, McMaster University, Hamilton, ON, Canada; 5 Department of Obstetrics and Gynaecology, University of British Columbia, Vancouver, BC, Canada; 6 Dr. Gilles Arcand Centre for Health Equity, Northern Ontario School of Medicine University, Thunder Bay, ON, Canada

**Keywords:** contraception, birth control, prison, incarceration

## Abstract

**Background:**

Women who experience incarceration lack equitable access and often face barriers to reproductive healthcare, including unmet needs for contraception. Previous studies have demonstrated that women who experience incarceration may want access to contraception and that providing contraception in prisons is feasible; however, it is unclear how this population wants to access contraception, and what their preference is for different forms of contraception.

**Objectives:**

We aimed to explore whether and how people with the capacity for pregnancy would like to access contraceptive education and services while incarcerated in provincial correctional facilities in Ontario.

**Design:**

We used a qualitative descriptive methodology using a qualitative content analysis approach.

**Methods:**

We conducted five focus groups with people aged 18–49 with the capacity for pregnancy and with current or prior experience of incarceration at an Ontario provincial correctional facility. Four groups were held at a provincial correctional facility for women, and one was held over Zoom for community-based participants.

**Results:**

Participants expressed a desire for contraception education and access during the incarceration period, including at intake, discharge, and throughout incarceration. They identified interests in accessible information about contraception, including through pamphlets, posters, and programs, and for care to be provided by a qualified women’s health clinician, who preferably was not employed by the jail. Participants felt that there were several barriers to accessing contraception while incarcerated, including long wait times, health care provider and other correctional staff attitudes, and lack of information on contraception options.

**Conclusion:**

This study identifies concrete strategies to address unmet needs for contraceptive care for women in custody. Study findings could inform the development of programs to support equitable contraceptive care access and reproductive health.

## Introduction

Women who experience incarceration lack equitable access and often face barriers to reproductive healthcare both in custody and in the community, contributing to unmet needs for contraception.^1–4^ In the community, barriers to contraception access identified by recently arrested women include economic, transportation, and clinic access factors.^2^ In custody, contraception may not be available: in a 2009 U.S. national survey, only 38% of 286 correctional healthcare providers reported that contraception was available in the correctional facilities where they worked.^3^ Even when available, contraceptive care in custody may not reflect best practices: a 2023 systematic review of contraceptive policies and practices found that 20 of 45 studies conducted in correctional facilities identified either outdated policies that were not in line with current best practices or no official policy detailing contraceptive care practices, while 12 studies identified that staff were hesitant to provide contraceptive care due to lack of education, training, and contraceptive knowledge.^4^ In quantitative studies in Rhode Island, USA and Ontario, Canada, over three quarters (84% and 77%, respectively) of incarcerated women reported having had an unplanned or unintended pregnancy, and the majority (72% and 80%, respectively) of those at risk for unintended pregnancy were not using reliable contraception.^1,5^ The lack of access to effective contraception contributes to unintended pregnancy,^1^ which is associated with a substantial morbidity and social burden, including increased maternal morbidity, adverse perinatal and child health outcomes, and excess medical costs.^6^ In addition, people may want to access contraception for other health-related reasons.^7^

Previous studies have demonstrated that women involved in the criminal justice system may want to access contraception care while incarcerated and that providing contraceptive services is feasible.^2,8–10^ However, it is unclear *how* women who are incarcerated want to access contraceptive services and contraceptive education, and there is limited evidence regarding preferences for forms of contraception.^2,9,11^ Information on the contraceptive needs and desires of incarcerated women could inform changes to correctional healthcare to support reproductive justice and women’s reproductive autonomy both during and after incarceration. In this study, we aimed to explore whether and how people with the capacity for pregnancy would like to access contraceptive services and education while incarcerated in provincial correctional facilities in Ontario.

## Methods

### Research team and reflexivity

All research team members were female and have experience in prison health research through this project and others. We approached this research with an explicit interest in reproductive justice for people in custody, including the rights to not have a child, to have a child, and to parent children in safe and healthy environments.^12^ The focus groups were facilitated by three of the study authors (J.G., E.N., J.Z.), who were a registered social worker, an obstetrics and gynecology resident, and an undergraduate student learner. The facilitators were not affiliated with the correctional facility and did not have a pre-existing relationship with any participants.

### Design

We used qualitative descriptive methodology, which aims for comprehensive reporting of the content under study using terms that are “accurate” to those used by participants, because our research question was straightforward and we wanted our analysis to hew closely to participants’ own words and experiences, using a qualitative content analysis approach.^13,14^ In this article, we used the Standards for Reporting Qualitative Research checklist to organize our reporting (see Supplemental Appendix A).^15^

### Terminology

For the purposes of this study, we use the term *woman/women* to refer to people who were currently or previously incarcerated in institutions designated for women. We recognize that gender-diverse individuals may not always be incarcerated in facilities that align with their gender identity, and that not all people who are incarcerated in facilities designated for women would self-identify as women. When referring to other research, we used the language used in each study, though we recognize terms for sex and gender may be conflated.

### Participants and setting

We conducted four focus groups at a provincial correctional facility for women in southern Ontario, Canada in December 2023, and one focus group over Zoom in February 2024 for community-based participants with recent experience of incarceration at an Ontario provincial correctional facility for women. Our inclusion criteria were: people aged 18–49 years who had been remanded or incarcerated in a provincial facility for women in Ontario (or on ranges (i.e. living units) for women in provincial correctional facilities that included men and women) in the last few years and had the capacity for pregnancy, that is, who could become pregnant on the basis of biological and behavioral factors. This included people who were biologically female, were or may later be sexually active with men, and had not had a hysterectomy. Our focus on people with the capacity for pregnancy reflects our interest in promoting and facilitating access to contraception for people at risk of unintended pregnancy, regardless of gender identity. We excluded anyone who did not fit these criteria.

In Canada, provincial correctional facilities hold people who are remanded (waiting for trial or sentencing) or sentenced to periods of incarceration <2 years,^10^ and in Ontario, provincial correctional facilities are publicly funded and governed by the Ministry of the Solicitor General. In provincial correctional facilities, hospitalizations and medically necessary physician services are paid for through the public health insurance system in Ontario. The Ministry of the Solicitor General pays for other healthcare services, for example, provides funding for nurses and social workers and for prescribed medications.

### Approvals

The study was reviewed and approved by the Hamilton Integrated Research Ethics Board (#7478) and the Ontario Ministry of the Solicitor General. The Ministry of the Solicitor General connected the research team to the Deputy Superintendent of the correctional facility designated for women to facilitate the research.

### Study recruitment

For the focus groups at the correctional facility, the Deputy Superintendent requested that the project team liaise with the Manager of Social Work Services to support project implementation. The Manager of Social Work Services supported the research team by ensuring information about the study was posted in the healthcare clinic as well as in the ranges for several days prior to the focus groups. Correctional staff handed out information about the study to people on ranges in which we would be conducting focus groups several days in advance of the focus group. The study information explained that people interested in participating could either submit a request to the Manager of Social Work Services or could indicate their interest to other correctional staff, who then conveyed this information to the Manager of Social Work Services.

For the focus group in the community, project team members shared information through professional and social networks, for example, through chapters of Elizabeth Fry Societies of Ontario, which are non-profit organizations that provide supports in custody and post-release to women and gender-diverse individuals involved in the Canadian justice system, and we invited people interested to contact a member of the project team by email to indicate their interest in participation. By email, we confirmed the date and time, provided a link for the virtual focus group, and provided the information and consent form.

For the focus group in the community, we compensated each participant with $50. We were not able to compensate participants in the focus groups in correctional facilities, due to Ministry of the Solicitor General policy.

Our sample size was determined by having as many participants as possible within logistical constraints. In the correctional facility, we were limited to conducting four focus groups, and we invited all those who expressed interest in each range for each of the four focus groups to participate. For the community-based recruitment, based on the project budget and timelines, as well as the expressions of interest in participation that we received, we conducted only one focus group. We judged the data to be sufficient to answer our question based on achieving appropriate “information power” through a relatively narrow study aim and a strong quality of dialogue.^16^

### Data collection methods

We decided to use focus groups both for feasibility and to inspire discussion in which participants could build on each other’s responses and reach different insights. The four focus groups at the provincial correctional facility took place in December 2023, and the focus group online on Zoom took place in January 2024. For the focus groups in the correctional facility, we were provided with a private room; only the facilitators and the participants were present in the room during the focus groups. The Zoom meeting was also private, with only the facilitators and participants in attendance.

Prior to the start of focus groups, the facilitators provided participants with information about the goals of the study and their positionality (as above) and obtained informed consent. For in-person focus groups at the correctional facility, facilitators collected written consent forms from participants. For the virtual focus group, we collected digitally signed consent forms via REDCap, a secure web application for building and managing online surveys and databases.^17^

For in-person focus groups, we started by asking participants to complete a brief demographic form on paper. For the virtual focus group, this form was sent via REDCap before the focus group. The demographic form included the participant’s nickname or first name, age, gender, race/ethnicity/Indigenous identity, and for those currently in custody, how long they had been in custody and for how long they expected to remain in custody (see Supplemental Appendix A).

In all focus groups, the facilitator or facilitators led the discussion based on a semi-structured focus group guide (see Supplemental Appendix B), which was developed with input and document review and critique from people with lived experience of incarceration, and included questions in four categories: birth control information needs, preferred ways to access information about birth control, preferred ways to access care related to birth control, and open-ended questions.

Focus groups were facilitated by one or two project team members (J.G., E.N., J.Z.; all female) who were all trained in qualitative research and had relevant expertise in prison health. Focus groups lasted between 21 and 47 min, with larger group size correlating with longer sessions. In-person focus groups were audio-recorded; virtual focus groups were video-recorded using Zoom recording, with participant consent. One facilitator for each focus group wrote field notes after the session.

### Data analysis

Audio data were transcribed in intelligent verbatim form, and de-identified by assigning each participant a unique code (e.g. Focus Group 1, Participant 1). We conducted a combined deductive and inductive qualitative content analysis approach including topic areas that had been identified a priori as question categories, and other topic areas arising from the data. We used Dedoose qualitative software (version 10.0.59), a cloud application for managing, analyzing, and presenting qualitative and mixed method research data (Los Angeles, CA, SocioCultural Research Consultants, LLC).^18,19^ Four coders (J.G., L.J., E.N., J.Z.; all female, including clinicians, qualitative researchers, student trainees, and a person with lived experience of incarceration) participated as analysts, recognizing that using coders with multiple perspectives creates opportunities for reflexivity during comparative analysis, which increases the credibility of findings.^20,21^ Each transcript was coded by two separate analysts and codes and themes were reviewed and iteratively developed in regular meetings with all analysts, alongside author F.G.K. for further perspectives.

For the article, we labeled quotes from participants in focus groups 1 to 4, which were conducted in custody, as FG (for focus group), followed by a number indicating the focus group and an additional participant number. For focus group 5, which was conducted in the community, we labeled quotes from participants as community focus group (for CFG) followed by the participant number.

## Results

Twenty-four people participated, including 19 (79.1%) who were incarcerated at the time of their focus group. The focus groups at the correctional facility ranged in size from three to six participants, and the community-based focus group had five participants. We include some self-reported demographics in Table 1 for readers to understand the participant pool of this study and potential generalizability to other contexts. Participants ranged in age from 23 to 46 years old, with a mean age of 35 (SD = 6.3). All respondents identified as women, though one participant did not respond to the question on gender. The most commonly reported race/ethnicity/Indigenous identity responses were White/European (62.5%), Black (29.2%), and Indigenous (20.8%). Most participants in custody (55%) had been in custody for 3 months or longer.

**Table 1.** Self-reported data for study participants (*N* = 24).

Variable	Category	*n* (%)
Race/ethnicity/Indigenous identity^a^ (*N* = 24)	White/European	15 (62.5%)
	Black	7 (29.2%)
	Indigenous	5 (20.8%)
	South American	1 (4.2%)
	Other	1 (4.2%)
	Don’t know	1 (4.2%)
Length of current incarceration (*N* = 20)	<3 months	9 (45%)
	3–<6 months	4 (20%)
	⩾6 months	7 (35%)
Expected length of time remaining in incarceration (*N* = 20)	<1 month	4 (20%)
	1–<3 months	5 (25%)
	3–<6 months	4 (20%)
	⩾6 months	7 (35%)

a Participants could choose multiple responses. Only categories selected by one or more participants are reported.

We identified six key topic areas in the data (see Table 2).

**Table 2.** Topic areas and method of identification.

Topic area	Description	Method of identification
Contraception information needs	Information about contraception information that participants said they or other women in prison need	A priori; focus group question category
Preferences for how to access information about contraception	How participants would prefer to access information about contraception	A priori; focus group question category
Preferences for how to access contraception	How participants would prefer to access contraception itself	A priori; focus group question category
Barriers to accessing contraception care in custody	Barriers that participants identified that impact whether and how they could access contraception care in custody	Arising from the data
Importance of accessing contraception care in custody	Importance of contraception care in custody that participants identified, given contraception access challenges on release	Arising from the data
Ways to improve access to contraception in custody	Participants’ suggestions for ways to improve access to contraception in custody	A priori; focus group question category

### Contraception information needs

Participants expressed interest in information about types of contraception available.

To see the options of different companies, and then to try which one you wanted. Because I know for example, there’s a needle form of one where you just get an injection. . . as opposed to taking an oral pill every day right at the same time. . . so you only have to go once every thirty days or so, as opposed to taking pills every day. Maybe you can’t remember medications. (FG4 P20)

They were also interested in how to use contraception, costs, purposes of contraception in addition to preventing pregnancy, and potential adverse side effects. One participant said:

My primary concern is how it [is] going to change me, is it going to make me more moodier, is it going to make me like, more snappy? . . .Is it going to make your mood swing, [are] your hormones going to change? Are you going to gain weight? . . .There [are] a lot of things that birth control does to your body that you don’t even predict. (FG4 P17)

### Preferences for how to access information about contraception

Participants discussed how they wanted to learn about contraception, from whom, using which media, and when. They described wanting to learn about contraception in various ways: in group sessions, “one-on-one” sessions for privacy, and in specific contraception clinics. Some participants felt information classes or group sessions would feel less intimidating and that it would be helpful to hear about others’ experiences and perspectives. Participants also suggested informational classes could be beneficial, in the context of a lack of programming in jails and a desire for more informational programming.

I think that it would be better to learn it in a group setting because everybody could talk and compare their experiences and stuff, so yeah, I think it would be better in a group setting. (FG4 P19)

They were also interested in having access to multiple strategies for accessible information.

Even if it was like starting in a group and then you had an opportunity to sign up for one-on-one if you have more in depth questions or more personal questions about your health. (FG3 P14)

Participants expressed an interest in having a dedicated contraception clinic, which may make appointments feel less rushed and support greater continuity of care.

Facilitated by a specific person, then you can always kind of go back to that person, then there’s no confusion or about who said what or you know, who agreed to what kind of thing. It’s just more accountability. (CFG1 P1)

Most participants agreed that they would prefer to access information on contraception from someone with specific contraception expertise and focus and who was female.

Yeah, like the nurse practitioner or the female nurse who knows how women’s bodies are. (FG3 P13)

They also described preferring that the person providing contraception information and care would be someone who was not on staff in the correctional facility.

Well actually I think that it shouldn’t be down to the staff I see how an option here is who would we like to get this information nurses or doctors in the jail, personally I think a public health or like a nurse specifically who is educated and to deal with women, I feel like it should be somebody from the community. (FG2 P9)

Regarding formats for accessing information on contraception, participants indicated several preferences. They suggested that physical pamphlets could be available throughout their incarceration, allowing them to access information whenever they wanted to. They suggested that pamphlets would also be a valuable addition to classes, so they could review the information discussed in sessions and so that the information could be accessible for people who were unable to attend informational sessions.

Something that we can read physically, see, hold, touch have something that we have some sort of information regarding the information we need for birth control would better our women’s health. (FG2 P9)

They also pointed out that posters are commonly used as a method of communication within jails and could also serve to inform correctional staff.

Pretty much just more like posters would be nice or like more advertising of [birth control] when you first come in, or when you leave, or even in the middle during your time. (FG3 P15)

Participants expressed interest in learning about birth control at all of the time points mentioned by facilitators. They identified the time of admission as a good opportunity to offer contraception.

When you’re first coming into intake, you want to see a nurse, a nurse practitioner, so maybe they can change that and offer us the options of going over birth control. (FG4 Participant Unidentified)

In addition, participants noted that information on contraception could be provided throughout their incarceration.

Sometimes people will be fed up about it, but if they had general info like how they have this study once a month or every couple weeks. . . hey you can come in and just look at different things, or if they had posters up and then it’s like hey ask to book an appointment . . . so that even if at first you turned it down, but then once you kinda detoxed a little bit and you settled in a bit and your mind clears a bit you can get used to where you are, and you may say okay now I want to address some issues that I may not have really noticed before because I have had everything going on. (FG3 P14)

Finally, they identified discharge planning as key opportunity to discuss contraception.

And ask them like, offer it to them. “You know, do you guys need birth control on your way out the door?.” (CFG1 P26)

### Preferences for how to access contraception

Participants described what wording should be used to ask about interest in birth control, when to talk about starting birth control, and when to start birth control. Participants recommended offering specific birth control clinics to which they could self-refer and having health care staff ask patients whether they are already on any type of contraception or whether they are interested in being on contraception. They also discussed the importance of being able to discuss other sexual health-related topics at the same time.

They should also have an option for, to talk to you about your STD testing and that kind of stuff [like] Pap tests. (FG1 P3)

Like with learning *information* about contraception as described above, participants suggested that the best time to be asked about *using* birth control would be both at the time of admission *and* prior to or during discharge planning.

Everyone has to do [admission], and everyone has to do discharge. So, if they had caught everybody with all these important questions. (FG1 P5)

Most participants said that the best time to start birth control was any time during their incarceration, and they described the benefits of starting birth control while incarcerated – while also ensuring there was a follow up plan after release to allow them to continue birth control.

People want to maybe start on birth control program in a place where you can consistently start taking it and get the best information, whereas maybe if you are on the street and all over the place it might be harder. Versus if you start in a routine, it might be easier to keep that routine when you get out, versus if you start on the street you know sometimes people don’t have access to health care and you would here. (FG3 P14)

### Barriers to accessing contraception care in custody

Participants cited difficulty accessing and discussing contraception care or any health care while incarcerated, lack of knowledge amongst correctional staff about available care, barriers to their ability to seek contraceptive care due to negative interactions with correctional staff, and difficulty with maintaining contraception during transitional periods, that is, between jail and community. Participants described long wait times to see physicians for any issue and feeling that appointments were often rushed and there wasn’t enough time to discuss contraception. Participants felt wait times were long due to a lack of healthcare providers, difficulty knowing how to request to see the physician, and appointments to discuss birth control either not being prioritized or patients being asked to put them off until discharge planning.

Like so, if we’re gonna have these kinds of issues with eyewear and with dental, you’re gonna run into the same problems with contraception. Because that’s going to be the last on their list. Because it’s not a necessity, they don’t believe it’s a necessity. (FG1 P3)

Participants discussed both a lack of staff availability and negative interactions with specific staff members that impeded their ability to seek contraception care. They also described how the lack of privacy from other staff members can make discussing contraception difficult.

I remember when I first came [onto the unit], and I remember asking one of the guards a question, and he said: “Just don’t even bother asking me, just ask the other inmates, if you want info.” And I remember I was like, whoa, you’re not even going to give me the rundown of how anything works? Or nothing? So, no wonder they’re not giving us any information on healthcare or programming, or anything like that, so. birth control is last on their list. Probably. (FG1 P5)

And just be stripped searched and everything else all the time and then you have the doctor and he doesn’t even apologize for the plastic forceps or the metal or whatever and it’s cold and you’re uncomfortable and not even say anything. (FG2 P6)

### Importance of accessing contraception care in custody

Participants discussed the cycle of leaving jail, getting pregnant, ending up re-incarcerated, or starting contraception then being incarcerated and stopping it or not having a way to access prescriptions after release.

Well I wasn’t planning on getting pregnant I was only out of jail for two months but I mean that is the whole thing a lot of women get out of jail and get pregnant right away. . . (FG2 P6)

They described how this cycle may be exacerbated by being given only a small amount of contraception on release.

. . .if you don’t have a doctor, you can get three days’ worth of birth control when you leave. If you’re on the pill. (FG1 P3)

Many participants highlighted the importance of addressing issues while incarcerated to reduce stressors and risk of pregnancy in the immediate post-release period. They reflected on how access to contraception could change this cycle.

For when you get released, you at one time or another we are all going to be out of jail, might be twenty years from now might be a year from now but at least we won’t have to worry about if you don’t want to have a baby right away or you want to get your life on track that’s a good thing to block that. (FG3 P13)

### Ways to improve access to contraception in custody

In addition to ways to improve access to contraception in custody described above, participants also discussed a desire for additional training for correctional officers.

I can understand you being a correctional officer, you’ve seen a lot of shit, but, at the end of the day, it’s person by person you have to deal with, you took an oath to be a person that is taking care of other people, essentially. You should absolutely think about this as if this was your child, as if this was somebody that you knew, because it just, it just makes that little bit of difference that makes us start to feel a little bit more like a human, being in there when people are showing us that they have your best interest at heart, a little thing, like how people take that for granted. (CFG1 P23)

They also hoped for more resources put toward birth control, including condoms and longer contraception access at release.

Yeah or. . .like book an appointment before you leave to get maybe the next three months of birth control covered. Yeah something like that, or six months depending on what you’re taking right?. (CFG1, P1)

## Discussion

This study identifies complex contraceptive needs and preferences among a sample of women with experience of incarceration, providing valuable insights for improving reproductive healthcare services in correctional facilities. Participants described a desire for better access to contraception and to comprehensive information about contraception, including the purposes, types, uses, and side effects. They indicated preferences for care provided by female healthcare professionals, dedicated contraceptive services, varied methods of information such as pamphlets, posters, and programming, and that information and care be provided by non-correctional health care staff. Participants wanted information about and access to contraception throughout incarceration, including at intake and prior to release.

Participants described existing barriers to healthcare access in custody, such as long wait times and negative interactions with healthcare providers and correctional officers. These experiences with staff included negative attitudes and communication challenges and were seen as significant obstacles to accessing care. This information reinforces the vital role that provider attitudes and communication skills play in building trust and improving healthcare access, particularly amongst marginalized groups.^9,22^

Our findings align with evidence from two qualitative studies conducted with formerly incarcerated women in New York City, in which nearly all participants felt that contraception should be available in correctional facilities, as incarceration presents an opportunity to access contraception, despite distrust in jail-provided medical care and systemic barriers to continuing contraception on release.^8,9^ Of note, in one of these studies many participants expressed lack of interest in birth control due to desire for pregnancy,^9^ a theme that did not come up within this study. Another study in Ontario, Canada found similar themes to both the New York study and the current study such as poor access to contraception, the potential of the correctional setting as an opportunity to start contraception, and contraception being included in both admission and release planning.^23^ Additionally, a systematic review examining contraception access amongst incarcerated women throughout the United States identified a similar interest in access to contraception, as well as similar barriers to care, including lack of information and lack of informed health care providers within institutions.^10^

The incarceration period provides a unique opportunity to provide reproductive care.^1,8–10^ However, the findings of our study, along with previous studies, highlight significant gaps in reproductive care for incarcerated women, underscoring the urgent need for systemic change. Equitable reproductive care access should meet the standards of care in the community, and reflect best practices for people in correctional facilities.^24^ For example, in the context of the high prevalence of traumatic experiences, including in custody, reproductive healthcare should be trauma-informed.^25,26^ This could include strategies such as staff training on trauma, universal screening upon health intake for trauma histories, being transparent and allowing as much autonomy as possible in healthcare decisions, and broader system level changes in policy and practice.^27^

### Limitations

This study has several limitations. Given the framing of the study as being about contraception, people who were not interested in contraception or felt their access was adequate may not have joined the study, though in the consent form we did state that we were interested in including people even if they were not interested in contraception (“We also want to figure out the best way to ask everyone about birth control, even if they do not need or want it”). The findings regarding interest in access to contraception information and care would likely not be generalizable to all women in custody. That notwithstanding, given our interest in reproductive justice and addressing unmet needs for contraception, we think a focus on this subset of the incarcerated population is appropriate. The focus groups in custody were conducted at a single correctional facility in Ontario, and those participants may have had different experiences and interests compared with people incarcerated in different facilities, both within Ontario and elsewhere. However, many of those participants would have experienced incarceration previously in other facilities, and we also conducted a focus group in the community, which would have included people who had been incarcerated at various correctional facilities. Therefore, the findings likely reflect experiences at multiple facilities and would therefore be generalizable beyond the single facility. Finally, due to budget limitations and resource constraints in the correctional facility, we conducted only four focus groups in custody and one focus group in the community, which may limit the breadth of the study’s findings. However, given that similar themes were highlighted across focus groups, including similar themes between the incarcerated and community focus groups, this does suggest that we did achieve saturation of themes.

### Implications for future research and practice

Further areas to explore in future research include which healthcare providers would be trusted by incarcerated women to provide health care, how contraceptive clinics could be integrated into the correctional healthcare system, and how to ensure continuity of contraceptive care from the time in custody through the transition back to the community, a concern that was voiced by participants in this study and previous studies.^8–10^ In addition, given historical and ongoing issues regarding reproductive coercion of incarcerated women, explicit attention should be focused on ways to enable and promote contraception access – in addition to reproductive justice more broadly – while simultaneously mitigating risks of reproductive coercion.^28^ Future initiatives could include piloting and evaluating some of the strategies described by participants, for example, a contraception clinic, designed in partnership with women who experience or have recently experienced incarceration.

## Conclusions

This study provides important insights into the contraceptive needs and preferences of incarcerated women in provincial correctional facilities. Addressing these needs will likely require a multifaceted approach, including targeted efforts to improve patient education, support diverse information delivery methods, develop dedicated contraceptive healthcare resources, and improve the quality of reproductive care within correctional settings. By working to better understand the needs and perspectives of incarcerated women, policymakers and healthcare providers can work toward ensuring equitable access to contraception and improving reproductive healthcare experiences in correctional environments.

## Supplemental Material

sj-docx-1-whe-10.1177_17455057261432612 – Supplemental material for Exploring access to desired and appropriate contraception during incarceration: A qualitative description of the experiences of women in custody in Ontario, Canada Supplemental material, sj-docx-1-whe-10.1177_17455057261432612 for Exploring access to desired and appropriate contraception during incarceration: A qualitative description of the experiences of women in custody in Ontario, Canada by Emily Norris, Jessica Gaber, Julia Zhu, Lindsay Jennings, Meredith Vanstone, Jessica Liauw, Jessica Jurgutis, Katherine E. McLeod and Fiona G. Kouyoumdjian in Women's Health

sj-docx-2-whe-10.1177_17455057261432612 – Supplemental material for Exploring access to desired and appropriate contraception during incarceration: A qualitative description of the experiences of women in custody in Ontario, Canada Supplemental material, sj-docx-2-whe-10.1177_17455057261432612 for Exploring access to desired and appropriate contraception during incarceration: A qualitative description of the experiences of women in custody in Ontario, Canada by Emily Norris, Jessica Gaber, Julia Zhu, Lindsay Jennings, Meredith Vanstone, Jessica Liauw, Jessica Jurgutis, Katherine E. McLeod and Fiona G. Kouyoumdjian in Women's Health
