# Supplementary material for: Exploring access to desired and appropriate contraception during incarceration: A qualitative description of the experiences of women in custody in Ontario, Canada
Source: Womens Health (Lond). 2026 Mar 13;22:17455057261432612. doi: 10.1177/17455057261432612 (PMC12988297; doi:10.1177/17455057261432612)
Supplement: sj-docx-2-whe-10.1177_17455057261432612 – Supplemental material for Exploring access to desired and appropriate contraception during incarceration: A qualitative description of the experiences of women in custody in Ontario, Canada [file sj-docx-2-whe-10.1177_17455057261432612.docx]

**Focus Group Guide**

| **Topic** | **Questions** |
| --- | --- |
| Information needs | Do you think women in jail want or need more information about birth control? If so, what kind of information do you think they would like?  What are some things that women in jail don’t know about birth control?  *Prompt:* What about things like what the different options are, how effective each option is, and common side effects? |
| Preferred ways to access information | What kinds of media would work best to help women in jail learn more about birth control? *Prompt:* For example, information sessions, pamphlets, informational videos, or something else.  Who would you prefer to learn about birth control from?  *Prompt:* For example, from nurses or doctors in the jail, a public health nurse, or someone with experience being in jail like a peer?  Would you prefer to learn about the options in a group setting or one-on-one? |
| Preferred ways to access care | How would you prefer to be asked about your interest in birth control? *Prompt:* For example, we could ask everyone on admission if they want to talk to a health care provider about birth control, or we could put up posters saying that women can submit a request if they want to talk about birth control.  At what point during your stay in the jail would you like to talk about birth control? *Prompt:* This could be when you’re admitted, during your stay, or right before release.  If talking about birth control in the clinic makes the most sense, would you prefer to talk about it during the regular clinic hours or during a special dedicated birth control clinic?  Are there other things that could be done to support women in the jail who want birth control in accessing it? |
| Open ended questions | Are there other things that you think we should know about how we can improve access to information on birth control and access to birth control for women in jail? |
